# Aggressive HIV-1?

**DOI:** 10.1186/1742-4690-2-13

**Published:** 2005-02-28

**Authors:** Ben Berkhout, Anthony de Ronde, Lia van der Hoek

**Affiliations:** 1 Department of Human Retrovirology, Academic Medical Center, University of Amsterdam, Meibergdreef 15, 1105 AZ Amsterdam, the Netherlands

## Abstract

New York City health officials announced on February 11, 2005 that a patient rapidly developed full-blown AIDS shortly after being diagnosed with a rare, drug-resistant strain of HIV-1. The New York City Department of Health issued an alert to all hospitals and doctors and a press conference was held to announce the emergence of an aggressive HIV-1 strain that may be difficult to treat and that appears to trigger rapid progression to AIDS. Is the panic justified?

## 

The two phenomena of rapid disease progression and multi-drug resistance, which are combined in the aggressive HIV-1 strain, are not unique. HIV-1 causes a persistent infection and this virus is generally not a fast killer. Within the Amsterdam Cohort Studies on HIV-AIDS, it takes on average 8.3 years from the time a person is first infected with HIV-1 for AIDS to develop, and another 17 months from AIDS to death [1]. However, the length of the incubation period varies from 2 months to more than 20 years. Cases where it takes much shorter are not uncommon (rapid-progressors), and likewise there is a significant group of socalled long-term non-progressors [2-5]. There seems uncertainty about the actual date of infection of the New York individual, such that AIDS may actually not have developed within 2 to 3 months, but rather within 20 months, which makes this case less exotic. Transmission of a drug-resistant HIV-1 variant is not uncommon either [6,7]. The number of cases have remained relatively small, but may be on the rise due to an increase in therapy failures [8]. The New York virus appeared resistant to three classes of antivirals (the Reverse Transcriptase inhibitors; nucleoside and non-nucleoside drugs, as well as Protease inhibitors), but this is not unexpected either in the era of combination-therapy in which therapy failure will usually mean the emergence of multi-drug resistant HIV-1 variants.

Drug-resistant HIV-1 variants usually have reduced replication capacity compared to a wild-type virus due to the mutations in the Reverse Transcriptase and Protease enzymes [9]. This loss in replication fitness may be even larger for a multi-drug resistant virus [10]. How does this relate to the aggressive disease course? More research is needed to resolve this issue. First, it is not always true that the acquisition of drug-resistance mutations causes a fitness loss. Even in case a loss is apparent, the virus may select compensatory changes over time, and the end result may in fact be a virus variant with increased replication fitness [11]. Second, one can only link a particular pathogenicity phenotype to a virus strain when a distinct disease pattern is seen in multiple infected persons. When an isolated case is discussed, it is equally possible that the particular disease pattern is not due to the virus, but rather due to a special property of the infected human host. Person-to-person variation in the immune system or other factors that interact with HIV-1 (receptors, innate immune factors etc) can greatly influence disease progression [12].

Overall, this case seems relatively rare but not necessarily alarming. Increased attention is not necessarily bad, but press conferences should be reserved for situations when a cluster of such transmissions is apparent. The current hype about super aggressive HIV-1 strains seems unfounded.

## Competing interests

The author(s) declare that they have no competing interests
